# Factors associated with self-reported discrimination against men who have sex with men in Brazil

**DOI:** 10.11606/S1518-8787.2017051000016

**Published:** 2017-11-13

**Authors:** Laio Magno, Inês Dourado, Luís Augusto V da Silva, Sandra Brignol, Ana Maria de Brito, Mark Drew Crosland Guimarães, Adele Benzaken, Adriana de A Pinho, Carl Kendall, Ligia Regina Franco Sansigolo Kerr

**Affiliations:** I Universidade do Estado da Bahia. Departamento de Ciências da Vida. Salvador, BA, Brasil; II Universidade Federal da Bahia. Instituto de Saúde Coletiva. Salvador, BA, Brasil; III Universidade Federal da Bahia. Instituto de Humanidades, Artes & Ciências Professor Milton Santos. Salvador, BA, Brasil; IV Universidade Federal Fluminense. Instituto de Saúde Coletiva. Niterói, RJ, Brasil; V Fundação Oswaldo Cruz. Instituto Aggeu Magalhães. Recife, PE, Brasil; VI Universidade Federal de Minas Gerais. Faculdade de Medicina. Departamento de Medicina Preventiva e Social. Belo Horizonte, MG, Brasil; VII Ministério da Saúde. Secretaria de Vigilância em Saúde. Departamento de IST, Aids e Hepatites Virais. Brasília, DF, Brasil; VIII Fundação Oswaldo Cruz. Instituto Oswaldo Cruz. Rio de Janeiro, RJ, Brasil; IX Center for Global Health Equit. Tulane School of Public Health and Tropical Medicine. New Orleans, Louisiana, USA; X Universidade Federal do Ceará. Departamento de Saúde Comunitária. Fortaleza, CE, Brasil

**Keywords:** Homosexuality, Male, Sexism, Risk Factors, Socioeconomic Factors, Gender and Health, Health Inequalities

## Abstract

**OBJECTIVE:**

To estimate self-reported discrimination due to sexual orientation among men who have sex with men (MSM) in Brazil and to analyze associated factors.

**METHODS:**

A cross-sectional study of 3,859 MSM recruited in 2008–2009 with respondent driven sampling. Data collection conducted in health centers in 10 Brazilian cities. A face-to-face questionnaire was used and rapid HIV and syphilis tests conducted. Aggregated data were weighted and adjusted odds ratio estimated to measure the association between selected factors and self-reported discrimination due to sexual orientation.

**RESULTS:**

The sample was predominantly young, eight plus years of schooling, *pardo* (brown), single, low-income, and identified themselves as gay or homosexual. The prevalence of self-reported discrimination due to sexual orientation was 27.7% (95%CI 26.2–29.1). Discrimination was independently associated with: age < 30 years, more years of schooling, community involvement and support, history of sexual and physical violence, suicidal thoughts, and unprotected receptive anal intercourse.

**CONCLUSIONS:**

The prevalence of self-reported discrimination among MSM in Brazil is high. These results challenge the assumptions that MSM-specific prevention and support programs are not required or that health professionals do not need special training to address MSM needs.

## INTRODUCTION

The prevalence of HIV infections among men who have sex with men (MSM) in various countries is disproportionately high^1^. In Brazil, the estimated prevalence of HIV infection between MSM above 18 years of age is around 14.2% (95%CI 12.1–16.6)^2^.

Among the factors that contribute to the continuation of elevated HIV levels among MSM, social determinants are a stand out, reflecting discrimination and the inequality of social, economic, organizational, and political power for many MSM populations. Combined with a range of risk factors such as sex without condoms, the number of partners, and difficult access to health services and diverse forms of prevention (e.g., Pre-Exposure Prophylaxis and Post-Exposure Prevention), MSM are especially vulnerable in this epidemic^4^. Studies have underlined that stigma and discrimination are among the main contributing factors to the continuation of the epidemic among MSM, often creating barriers to prevention, testing, and treatment services for HIV^5^.

Another aggravating factor is that homoerotic practices are deemed illegal in 78 countries in the world and, in five countries, are punishable by death^6^. Although homosexuality is not illegal in Latin America and the Caribbean, discrimination is a serious problem^7^. In Brazil, according to the Secretary of Human Rights of the Presidency of the Republic, in 2012, there were around 27 notifications per day of homophobic incidents – and this is assumed to be underreported^8^.

This study revisited the first national HIV surveillance survey among MSM in Brazil to estimate discrimination due to sexual orientation amongst MSM, and to analyze potential associated factors.

## METHODS

This study used data from the national study “Behavior, Attitudes, Practices, and Prevalence of HIV and Syphilis amongst Men who Have Sex with Men”, conducted in 10 Brazilian cities (Manaus, Recife, Salvador, Campo Grande, Brasília, Curitiba, Itajaí, Santos, Belo Horizonte, and Rio de Janeiro) between October 2008 and October 2009. Its main objectives were to study the behavior, attitudes, and sexual practices of MSM and estimate the prevalence of HIV and syphilis. The total population was 3,859 men who reported at least one sexual contact with another man in the previous 12 months. Other criteria for inclusion in the study was being 18 years or over, not identifying as transgender, residing in the cities selected in the study, and signing the informed consent form.

In each site the sample was recruited utilizing respondent driven sampling (RDS)^9^ ^,^ ^10^. This is a chain link sampling method that begins with a convenience sample of members of the target population called “seeds”. Initially, formative research was undertaken in each city, which consisted of semi-structured interviews and focus groups. Subsequently, about six MSM (nominated seeds), with relatively large contact networks characterized by a diverse age range and socioeconomic makeup were chosen by the researchers in each city. A total of 140 seeds were used across the sites. Each participant received three coupons to recruit participants to the study. This process was repeated until the sample size was reached (n = 3,859). Participants received a primary incentive of R$ (Brazilian Real 2009) 15.00 (US$10.00) and an incentive of R$(Brazilian Real 2009) 10.00 (US$6.67) for each of their recruits who completed the survey. Data collection took place in the health centers of City Health Departments. A face-to-face questionnaire was used and included questions concerning the size of the social networks of the contacts; sociodemographic characteristics; identity and sexual behavior; use of alcohol and other drugs; mental health; self-reported discrimination (for sexual orientation, race, social condition, or age), self-reported history of violence (verbal, physical, sexual); social integration and participation; information on sexuality transmitted diseases (STD), access to condoms; health care and STD symptoms.

After the interview, participants were offered a rapid HIV test, using the following tests: Rapid Check HIV-1&2 (Center for Infectious Diseases, Universidade Federal do Espírito Santo, Vitória, Brazil) and Bio-Manguinhos HIV-1&2 (Institute for Immunobiological Technology, Fundação Oswaldo Cruz, Rio de Janeiro, Brazil). All participants received pre-and post-test counseling, educational material, and condoms. Those who tested positive for HIV were referred to specific health centers for HIV care. The research project was approved by the National Ethics Commission of Brazilian Research (CONEP 14494). Complete details of the overall study have been reported previously^2^.

### Outcome and Other Study Variables

The main outcome variable of this study is self-reported discrimination due to sexual orientation and it was defined according to the following question: “In the last 12 months, did you feel discriminated against by some person or institution because of your sexual orientation?” Other variables included in the analysis were: a) sociodemographic: age, self-reported skin color, conjugal situation, sexual orientation; b) economic and educational status: monthly income and years of education; c) history of violence: having been forced to have sex (sexual violence), experience of physical violence, and being verbally threatened or humiliated; d) access to health care and social support: STD counseling in the last 12 months, community involvement and support; e) mental health: feeling sad or depressed (never; at least once) in the last six months, had suicidal thoughts in the last six months; f) risk factors for STD: unprotected receptive anal intercourse with casual and steady male partners in the last six months.

### Analysis

As shown in Table 1, there was no indication of homophily with regard to the outcome of interest (self-perceived discrimination) in any of the 10 cities (ranging from 0.955 to 1.125, median = 1.04). The rationale for data analysis was to use statistical method in order to take into account the dependence among observations, resulting from the recruitment chains and the unequal probabilities of selection and from the different sizes of networks of each participant. The probability that an individual participates in RDS research depends on the size of their social network^9^. To generate a total for the sample, data from each survey were weighted by the inverse probability of individual selection, proportional to the size of the social network reported by each respondent. The procedure is fully described in Kerr et al.^2^

**Table 1:** Prevalence of self-perceived discrimination of men who have sex with men in 10 cities. Brazil, 2009.

Cities	Unadjusted (%)	95%CI	Weighted* (%)	95%CI	Homophily
Manaus	36.4	33.1–39.6	29.3	26.2–32.4	1.030
Recife	47.0	41.6–52.2	39.3	34.1–44.4	1.064
Salvador	34.1	29.3–38.9	26.6	22.1–31.0	1.019
Brasília	41.8	36.3–.47.2	39.3	33.8–44.7	0.992
Campo Grande	32.3	27.3–37.2	25.9	21.3–30.5	1.125
Belo Horizonte	40.8	34.8–46.7	34.0	28.2–39.7	1.046
Rio de Janeiro	31.4	26.3–36.4	23.3	18.6–27.9	1.041
Santos	23.2	17.6–28.7	20.4	15.0–25.6	1.043
Curitiba	33.2	28.0–38.4	23.9	19.2–28.6	0.955
Itajaí	21.5	16.6–26.2	12.5	8.6–16.3	1.086

Total	34.8	33.2–36.3	27.7	26.2–29.1	-

* Weighted prevalence according to the size of the social network and the proportion of MSM in each city.

Participants personal social network size and the resulting weight were measured using the question: “How many MSM were 18 or older had people with whom they were familiar and who they might invite to participate in this study”. Moreover, as the analysis was conducted considering the 10 cities simultaneously, the sample was also weighted by the relative population size of Brazilian men, 18–64 years of age, in each site, and the proportional size of the MSM population in each municipality^11^ and considering each municipality as a stratum. This method of analyzing RDS dataset was proposed by Szwarcwald et al.^12^ and published studies applied this method^13^ ^,^ ^14^. The method proposed here considers both the chain-link effects and the unequal selection probabilities to estimate the prevalence rate, the standard error and 95%CI, and the design effect. The 10-city dataset was then analyzed using logistic regression wherein each city was treated as a stratum.

Multivariate analysis involved: 1) a review of the literature to identify factors consistently identified in studies of discrimination due to sexual orientation; 2) bivariate analyses were conducted to identify additional variables for inclusion in the multivariate analysis. Adjusted odds ratio (OR) and 95%CI were estimated to measure the association between selected factors and self-reporting of discrimination due to sexual orientation. Estimates with a p < 0.10 were selected for a weighted logistic regression analysis, which began with a saturated model and backward elimination that progressively removed variables until an adequate model of estimation was selected. Data was analyzed using Stata®, version 12 (Stata Corp, College Station, Texas, USA).

## RESULTS

A sample of 4,048 MSM were initially recruited in 10 Brazilian cities – 188 (4.46%) were considered ineligible and one refused to participate –, for a total of 3,859 participants. Of these, 3,635 MSM (94.2%) provided an answer to the discrimination question and were included in this analysis.

The overall prevalence of self-reported discrimination was 27.7% (95%CI 26.2–29.1), ranging from 21.5% in Itajaí to 47% in Manaus (Table 1). Location of the discrimination included the street environment (13.7%), shopping areas (6.9%), entertainment locations (6.4%), school or university (5.9%), religious gatherings (3.7%), and in health services (1.3%). Participants were mostly young (60.5%), had a level of education of 8–14 years (57.4%), self-identified as *pardo* (53.8%), were single (84.4%), and living on a monthly income (47.6%) of less than one Brazilian minimum wage (R$465 = US$230 in 2009). They identified themselves as gay or homosexual (50.5%), bisexual (30.3%), MSM (11.4%), and heterosexual (7.7%). Almost a third (27.2%) reported unprotected receptive anal intercourse (URAI) with casual male partners in the last six months and almost half (47.5%) reported URAI with steady male partner in the last six months. More than half reported consistent condom use with casual male partners during the past year (72.8%), while 52.5% reported condom use with steady partners. Half of the participants reported having been subjected to some kind of violence: sexual (14.6%), verbally threatened or humiliated (42.3%), or physical violence (15.9%). Few received STD counseling in health settings (11.8%). Feeling sad or depressed during the last six months and having suicidal thoughts during the last six months was reported by 14.6% and 19.7%, respectively. The majority reported low or moderate consumption of alcoholic beverages (36.1% and 49.5% respectively). And few (9.9%) reported community involvement and support (Table 2).

**Table 2:** Distribution of characteristics of the 3,635 MSM (men who have sex with men) participants of the study in 10 Brazilian cities.

Characteristic	n^a^	% Weighted^b^
Self-reported discrimination in the last 12 months		
Yes	1,266	27.7
No	2,369	72.3
Age		
≤ 30 years	2,602	60.5
> 30 years	1,033	39.5
Years of education		
0–7 years	727	28.4
8–14 years	2,338	57.4
≥ 15 years	570	14.2
Skin color/ethnicity		
White	1,011	26.2
Black	458	12.6
*Pardo* (Brown)	2,047	53.84
Yellow/Indigenous	119	3.0
Conjugal situation		
Married or lives with companion	592	16.3
Single/Widowed/Divorced/Separated	3,043	83.7
Monthly income		
≤ 1 minimum wage	1,666	47.6
≥ 2 minimum wage	1,969	52.4
Sexual orientation		
Heterosexual	281	7.7
Gay/Homosexual	1,836	50.5
Bisexual	1,103	30.3
MSM	415	11.4
URAI with casual male partners in the last 6 months		
Yes	567	27.2
No	1,597	72.8
URAI with steady male partners in the last 6 months		
Yes	828	47.5
No	884	52.5
Counseling about STD in health service		
Yes	595	11.8
No	3,040	88.2
Verbal violence		
Yes	1,652	42.3
No	1,983	57.7
Physical violence		
Yes	539	15.9
No	3,096	84.1
Sexual violence		
Yes	576	14.6
No	3,059	85.4
Felt sad or depressed in the last 6 months		
No or infrequently	3,159	80.3
Frequently	475	19.7
Suicidal thoughts in the last 6 months		
No or infrequently	3,094	80.1
Frequently	475	19.9
Use of alcohol		
Low	1,461	36.1
Moderate	1,849	49.5
High	323	14.4
Community involvement and support		
Yes	395	9.9
No	3,240	90.1

URAI: unprotected receptive anal intercourse; STD: sexuality transmitted diseases

^a^ Excluding missing data.

^b^ Weighting calculated according to the size of the social network and the proportion of MSM in each city.

In bivariate analysis, the following factors were identified with the self-reporting of discrimination due to sexual orientation: age less than or equal to 30 years, 8–14 and ≥ 15 years of education, identifying as gay or homosexual, bisexual or MSM, URAI with casual male partners in the last six months, URAI with steady male partner in the last six months, not having received counseling for STD in health settings, feeling sad or depressed during the last six months, suicidal thoughts during the last six months, and reported community involvement and support (Table 3).

**Table 3:** Bivariate analysis of factors associated with the self-reporting of discrimination due to sexual orientation among men who have sex with men (MSM) in Brazil.

Variable	wOR*	95%CI	p
Age (years)			
≤ 30	1.00		
> 30	1.84	1.59–2.17	< 0.001
Years of education			
0–7 years	1.00		
8–14 years	2.68	2.21–3.25	< 0.001
≥ 15 years	2.86	2.23–3.67	< 0.001
Skin color/ethnicity			
White	1.00		
Black	0.79	0.63–1.00	0.06
*Pardo* (Brown)	0.92	0.77–1.09	0.3
Yellow/Indigenous	0.75	0.48–1.20	0.2
Conjugal situation			
Married or lives with companion	1.00		
Single/Widowed/Divorced/Separated	0.84	0.68–1.02	0.09
Monthly income			
≤ 1 minimum wage	1.00		
≥ 2 minimum wage	0.93	0.80–1.08	0.4
Sexual orientation			
Heterosexual	1.00		
Gay/Homosexual	14.70	9.02–23.97	< 0.001
Bisexual	3.82	2.30–6.34	< 0.001
MSM	3.39	1.99–5.76	< 0.001
URAI with casual male partners in the last 6 months			
No	1.00		
Yes	1.38	1.12–1.70	< 0.001
URAI with steady male partners in the last 6 months			
No	1.00		
Yes	1.97	1.58–2.45	< 0.001
Counseling about STD in health service			
Yes	1.00		
No	1.53	1.23–1.89	< 0.001
Verbal violence			
No	1.00		
Yes	6.62	5.62–7.80	< 0.001
Physical violence			
No	1.00		
Yes	3.98	3.26–4.86	< 0.001
Sexual violence			
No	1.00		
Yes	2.96	2.45–3.58	< 0.001
Felt sad or depressed in the last 6 months			
No or infrequently	1.00		
Frequently	1.47	1.23–1.75	< 0.001
Suicidal thoughts in the last 6 months			
No or infrequently	1.00		
Frequently	1.48	1.24–1.76	< 0.001
Use of alcohol			
Low	1.00		
Moderate	0.94	0.81–1.10	0.5
High	0.58	0.45–0.74	< 0.001
Community involvement and support			
No	1.00		
Yes	2.73	2.19–3.41	< 0.001

URAI: unprotected receptive anal intercourse; STD: sexuality transmitted diseases

* Weighted odds ratio calculated according to the size of the social network and the proportion of MSM in each city.

In the final weighted multivariate logistic model (Table 3), MSM who were 30 years of age or younger had over two times greater odds of self-reporting discrimination than older subjects (OR = 2.45, 95%CI 1.88–3.20). MSM with higher levels of education – eight to 14 years (OR = 2.02, 95%CI 1.41–2.88) or more than 15 years (OR = 1.80, 95%CI 1.19–2.27), had a greater chance of self-reporting discrimination due to sexual orientation compared with those with less than seven years of education.

The odds of self-reporting discrimination due to sexual orientation was greater among MSM who reported experience of physical violence (OR = 2.97, 95%CI 2.154–4.10), sexual violence (OR =2.21, 95%CI 1.63–3.00), had frequently suicidal thoughts over the last six months (OR = 1.42, 95%CI 1.04–1.94), or had reported community involvement and support (OR = 1.70, 95%CI 1.21–2.40) than among those that did not report these experiences. Furthermore, MSM who reported URAI with steady male partners in the last six months had a greater chance of self-reporting discrimination due to sexual orientation than those that reported consistent condom use (OR = 1.89, 95%CI 1.50–2.39) (Table 4).

**Table 4:** Analysis by logistic regression of factors associated with self-reporting of discrimination due to sexual orientation amongst men who have sex with men (MSM) in Brazil.

Variable	wOR (95%CI)*	p
Age (years)		
≤ 30	1.00	
> 30	2.45 (1.88–3.20)	< 0.001
Years of education		
0–7 years	1.00	
8–14 years	2.02 (1.41–2.88)	< 0.001
≥ 15 years	1.80 (1.19–2.72)	0.005
URAI with steady male partners in the last 6 months		
No	1.00	
Yes	1.89 (1.50–2.39)	< 0.001
Physical violence		
No	1.00	
Yes	2.97 (2.15–4.10)	< 0.001
Sexual violence		
No	1.00	
Yes	2.21 (1.63–3.00)	< 0.001
Suicidal thoughts in the last 6 months		
No or infrequently	1.00	
Frequently	1.42 (1.04–1.94)	0.02
Community involvement and support		
No	1.00	
Yes	1.70 (1.21–2.40)	< 0.001

URAI: unprotected receptive anal intercourse

* Weighted odds ratio calculated according to the size of the social network and the proportion of MSM in each city.

## DISCUSSION

Prevalence of self-reporting discrimination due to sexual orientation was as high in this study as encountered in countries where homosexuality is criminalized, such as Namibia, Botswana, and Malawi, countries that report levels of discrimination of 24.7%, 27.4%, and 19.1%, respectively^15^. The prevalence encountered in our study was similar to or greater than that reported in many cities in the United States of America. In Phoenix, Albuquerque, and Austin, 11.2% of young gay and bisexual men from those three cites reported experiencing of discrimination^16^. In San Francisco, 30.1% reported one or more experiences of discrimination because of sexual orientation: 9.6% were assaulted, 4.1% were discriminated against professionally, and 16.4% had been discriminated against personally^17^. In New York and Los Angeles, a study undertaken with Latino MSM estimated a rate of homophobia in the last 12 months of 17% and 14%, respectively^18^.

In our study, the youngest subjects reported a greater prevalence of discrimination due to sexual orientation than older MSM. This is similar to findings such as the study cited above in the USA, where the chance of suffering verbal abuse and physical violence was greater among MSM younger than 21 years of age, in comparison to older MSM^16^. Another study in five countries showed that younger MSM had a greater perception of discrimination and internalized homophobia, in comparison with older MSM, and had less access to HIV preventive services^19^. Several factors may account for this phenomena: discrimination may be voiced more frequently by younger heteronormative populations, younger populations of MSM may be more open about their identities or more willing to see microaggressions as discrimination, or psychological compensation may be occurring in older MSM: repeated exposure to abuse in early phases of life may result in psychological desensitization and in the creation of cognitive mechanisms that help support or neutralize the effects of abuse^20^.

Recently in Brazilian society, new forms of gay sociability are being seen including interaction through the internet and “apps”^21^, as well as more open relationships between younger gays and their families. New studies are required to better understand the effects of these changes in the field of health, as well as exploring responses to prejudice and discrimination that improve health of the entire population.

Discrimination can significantly affect the mental health of gays and other MSM^16^ ^,^ ^17^ ^,^ ^22^. Our results show that MSM who frequently experienced suicidal thoughts had greater odds of self-reporting discrimination due to sexual orientation. In a study undertaken in the USA, people with experiences of discrimination and physical violence were two times as likely to have suicidal thoughts^16^ when compared to those who had not suffered these experiences. In a study in Tanzania, MSM who suffered moral and verbal abuse showed the highest levels of depression^22^. In another study using the 2009 RDS dataset, MSM who experienced sexual violence, independently of the effect of other variables, had a two times greater odds of reporting suicidal thoughts than those not reporting^14^. A study carried out in Lesotho showed that depression was positively associated with: feeling of rejection by friends because of sexual orientation, being verbally discriminated because of homosexuality, fear to walk in public places, and history of blackmail^23^.

Our study showed that MSM that reported URAI with steady male partners had a greater chance of self-reporting discrimination due to sexual orientation, when compared with those who always used condoms. Other studies indicate that the experience of discrimination can increase the chance of exposure to riskier sexual behaviors, such as unprotected anal sex^17,18,24–26^. In another study using RDS conducted in 2009, various factors were independently associated with unprotected anal sex: living with a male partner, illicit drug use, having a stable partnership, having sex with men only, having few friends encouraging condom use, and high self-perceived risk for HIV infection^13^.

The literature has also indicated that the effects of stigma and discrimination can be an important barrier for MSM to access health services^4^, diminishing the possibility for HIV testing^27^. It is important to emphasize that after more than three decades of the HIV/AIDS epidemic, fear of going to an HIV/AIDS reference center and being recognized by others as HIV positive is still reported^5^ ^,^ ^28^. Furthermore, we still encounter in the literature reports that HIV infection among MSM is justified as an example of “deserving it”, a type of punishment due to immoral behavior^28^. Other negative images that can act as barriers to health care services access by MSM is the idea of homosexuals as “social deviants, or adepts of dangerous and reprehensible practices”^21^. Regarding these social markers or values associated with homosexual practices, Silva^29^ calls attention to how unprotected sexual practices is interpreted differently: while heterosexuals report unprotected sex as “only” a search for greater sexual pleasure, they interpret unprotected sex among MSM as a risk taking behavior. On one hand, heterosexuality is considered to be within normality and, on the other hand, homosexuality happens on the margins, understood as “strange”, “stupid” or “pathological”^29^.

In our study, the chance of reporting discrimination due to sexual orientation was greater among MSM who did not receive counseling for STD in health care settings. We cannot establish a causal relationship of these factors, but we can hypothesize that such self-reporting can be a maker of potential discrimination in health services in Brazil^30^.

In terms of limitations, interpretation of RDS data remains controversial, but it does improve on snowball sampling by generating longer chains, and can be operationally systematic and rigorous. Here, when used to generate a national-level sample, a further limitation is that that “RDS samples are not designed to be merged, and are best analyzed on a site by site basis. Aggregating the independent networks to generate a single sample violates an assumption of RDS that a sample forms one complete network component. A related limitation is that the 10 cities selected may not represent MSM in Brazil. The cross-sectional design does not permit the identification of a temporal association between exposure and outcome; therefore, we do not know if the self-reporting of discrimination owing to sexual orientation is a result of the analyzed variable or if these are a consequence of the discrimination. Results of RDS studies should avoid extrapolations to other settings.

In terms of strengths, in our study, whereas other sampling methods were without sampling frames, RDS was one of the methods available to assess hard-to-reach groups, which may bring concrete gains, as was shown in a comparison of RDS and other sampling methods^31^.

It is important to remember that, while Brazil does not possess laws that criminalize homosexuality, Brazilian culture is marked by *machismo* (male chauvinism) and patriarchalism^6^, which, in many situations, exposes MSM to stigma, discrimination, and violence. In our study, we observed that physical and sexual violence were associated with self-reporting of discrimination due to sexual orientation, indicating that the experiences of violence can be understood by MSM as outcomes consequent to discrimination. Sabidó et al.^14^ also found similar results between sexual violence and self-reporting of discrimination.

Our study also shows that MSM who reported community involvement and support had a greater likelihood of self-reporting discrimination. A previous study, undertaken with 406 MSM who reported community involvement and support showed that MSM who self-report discrimination had a 50% higher chance of participating in these groups^32^. It is likely that membership either exposes MSM to discrimination or enhances MSM willingness to report discrimination.

Although Lesbians, Gays, Bisexuals and Transgender social movements in Brazil are long-lasting and well-established, Brazil has still not developed legal mechanisms to respond to discrimination against sexual minority populations. Furthermore, current religious, political, and social movements in Brazil appear to be moving in an opposite direction, leading to a still greater increase in discrimination and stigma (such as the recent prohibition of educational videos and material regarding comprehensive sexual education in Brazilian schools^33^). Concerns for MSM health, including HIV/AIDS mandate re-mobilizing government and civil society to both restore the status quo ante, and to more broadly defend human rights for the social and physical protection of minority populations. To not do so diminishes the health of us all.
